# Licochalcone A specifically induces cell death in glioma stem cells via mitochondrial dysfunction

**DOI:** 10.1002/2211-5463.12226

**Published:** 2017-05-08

**Authors:** Kenta Kuramoto, Shuhei Suzuki, Hirotsugu Sakaki, Hiroyuki Takeda, Tomomi Sanomachi, Shizuka Seino, Yoshitaka Narita, Takamasa Kayama, Chifumi Kitanaka, Masashi Okada

**Affiliations:** ^1^ Department of Molecular Cancer ScienceYamagata University School of MedicineJapan; ^2^ Department of Clinical OncologyYamagata University School of MedicineJapan; ^3^ Department of Obstetrics, GynecologyYamagata University School of MedicineJapan; ^4^ Research Institute for Promotion of Medical SciencesFaculty of MedicineYamagata UniversityJapan; ^5^ Department of Neurosurgery and Neuro‐OncologyNational Cancer Center HospitalTokyoJapan; ^6^ Department of NeurosurgeryYamagata University School of MedicineJapan; ^7^ Department of Advanced Cancer ScienceFaculty of MedicineYamagata UniversityJapan

**Keywords:** apoptosis, cancer stem cells, crude drugs, glioblastoma, liquorice, respiratory chain

## Abstract

Glioblastoma multiforme is the most malignant primary intrinsic brain tumor. Glioma stem cells (GSCs) are associated with chemoradiotherapy resistance and the recurrence of glioblastomas after conventional therapy. The targeting of GSCs is potentially an effective treatment for the long‐term survival of glioblastoma patients. Licochalcone A, a natural chalconoid from licorice root, exerts anticancer effects; however, its effect on GSCs remains unknown. We found that Licochalcone A induced massive caspase‐dependent death in GSCs but not in differentiated GSCs nor normal somatic and neural stem cells. Prior to cell death, Licochalcone A caused mitochondrial fragmentation and reduced the membrane potential and ATP production in GSCs. Thus, Licochalcone A induces mitochondrial dysfunction and shows promise as an anticancer stem cell drug.

**Abbreviations**
ABC: ATP‐binding cassette
ACADVL: acyl‐CoA dehydrogenase, very long chain
CCCP: carbonyl cyanide 3‐chlorophenylhydrazone
GAPDH: glyceraldehyde‐3‐phosphate dehydrogenase
GFAP: glial fibrillary acidic protein
GSCs: glioma stem cells
Lico A: Licochalcone A
PARP: poly(ADP‐ribose) polymerase
RT: room temperature

Glioblastoma multiforme is a Grade IV astrocytoma according to the World Health Organization classification, and it is the most malignant primary brain tumor with a 5‐year survival rate of approximately 5% 1. Cancer stem cells, including glioma stem cells (GSCs), are a small subpopulation of tumors that possess stem‐like properties and the capacity for tumor initiation. It is widely accepted that tumors are hierarchically organized by various degrees of differentiating cancer cells, and the top of the hierarchy is occupied by cancer stem cells 2, 3. Indeed, residual cancer stem cells after clinical treatment, including conventional chemotherapy, are regarded as the cause of secondary tumor development and cancer recurrence 4. A growing body of evidence supports the existence of cancer stem cells in various human cancers, including glioblastoma multiforme 5.

Licorice root has been used worldwide for thousands of years as a traditional drug for gastric ulcers, bronchial asthma, and inflammation 6. Licochalcone A (Lico A) is a major chalcone extracted from the root of Xinjiang licorice, *Glycyrrhiza inflata* 6. Lico A possesses multiple pharmacological effects, such as antiparasitic activity for leishmania 7 and malaria 8, anti‐inflammatory activity 9, and anti‐obesity activity 10, as well as antitumorigenic effects 11, 12, 13. Lico A induces apoptosis in gastric, cervical and oral cancer 11, 13, 14; however, the effects of Lico A on cancer stem cells remain unknown. We examined the effect of Lico A on GSCs and glioma cells that differentiated from GSCs. Lico A strongly induced caspase‐dependent cell death in GSCs but not in differentiated GSCs. Cell death was a consequence of caspase‐9 activation and a reduction in the mitochondrial membrane potential in GSCs. This GSC‐specific response targeting mitochondria suggests that Lico A or its active compounds have a therapeutic potential against glioblastoma multiforme.

## Materials and methods

### Reagents and antibodies

Licochalcone A (Lico A), carbonyl cyanide 3‐chlorophenylhydrazone (CCCP), and antimycin A were purchased from Sigma (St. Louis, MO, USA). Z‐VAD‐FMK was purchased from Peptide Institute, Inc. (Osaka, Japan). Lico A, CCCP, antimycin A, and Z‐VAD‐FMK were dissolved in dimethyl sulfoxide to prepare a 10 mm stock solution. Anti‐Caspase‐3 (#9662), anti‐Caspase‐9 (#9502), anti‐Caspase‐8 (#9746), anti‐poly(ADP‐ribose) polymerase (PARP) (#9542), anti‐Sox2 (#3579), anti‐glial fibrillary acidic protein (GFAP) (#3670), anti‐c‐Jun (#9165), anti‐phospho‐c‐Jun (Ser 63) (#9261), and anti‐glyceraldehyde‐3‐phosphate dehydrogenase (GAPDH) (#5174) antibodies were purchased from Cell Signaling Technology, Inc. (Danvers, MA, USA). The anti‐acyl‐CoA dehydrogenase, very long chain (ACADVL) antibody was kindly gifted by T. Osumi (University of Hyogo) 15, 16. Horseradish peroxidase (HRP)‐conjugated secondary antibodies and an Alexa488‐conjugated secondary antibody were purchased from Jackson ImmunoResearch (West Grove, PA, USA) and Thermo Fisher Scientific (Waltham, MA, USA), respectively.

### Cell culture

The GSCs used in this study (GS‐Y01, GS‐Y03, U87GS, GS‐NCC01, A172GS) were previously described 17, 18. These GSC lines were maintained under monolayer stem cell culture conditions 17, 18, 19. Briefly, cells were cultured on collagen‐I‐coated dishes (IWAKI, Tokyo, Japan) in stem cell culture medium (DMEM/F‐12 supplemented with 1% B27 [Thermo Fisher Scientific], 20 ng·mL^−1^ EGF and FGF2 [Peprotech, Inc., Rocky Hill, NJ, USA], d‐(+)‐glucose [final concentration 26.2 mm], l‐glutamine [final concentration 4.5 mm], 100 units·mL^−1^ penicillin and 100 μg·mL^−1^ streptomycin). Rat cortical neural stem cells (NSCs) were purchased from R&D systems (Minneapolis, MN, USA) and cultured on Geltrex (Thermo Fisher Scientific)‐coated dishes in the stem cell culture medium 20. The stem cell culture medium was changed every 3 days, and EGF and FGF2 were added to the stem cell culture medium every day. Differentiation of GSCs was induced by culturing cells in DMEM/F‐12 medium supplemented with 10% FBS (Sigma), 100 units·mL^−1^ penicillin, and 100 μg·mL^−1^ streptomycin for 2 weeks. Normal human IMR‐90 fetal lung fibroblasts were purchased from the American Type Culture Collection (Manassas, VA, USA) and maintained in DMEM medium supplemented with 10% FBS, 100 units·mL^−1^ penicillin, and 100 μg·mL^−1^ streptomycin. All experiments that used rat NSCs or IMR‐90 cells were performed using low‐passage numbers (less than 4 and 8, respectively).

### Cytotoxicity assay

Viable and dead cells were identified by their ability and inability to exclude vital dyes, respectively 19, 21, 22. Briefly, cells were treated with drugs as described in the figure legends, then cells were stained with 0.2% trypan blue for 1 min at room temperature (RT), and the number of viable and dead cells was determined using a hemocytometer. The percentage of dead cells was defined as 100 × (number of dead cells/[the number of viable + dead cells]).

### Immunoblot analysis

Cells were washed with ice‐cold phosphate‐buffered saline (PBS) and lysed in RIPA buffer (10 mm Tris‐HCl [pH 7.4], 0.1% SDS, 0.1% sodium deoxycholate, 1% Nonidet P‐40, 150 mm NaCl, 1 mm EDTA, 1.5 mm sodium orthovanadate, 10 mm sodium fluoride, 10 mm sodium pyrophosphate, and protease inhibitor cocktail set III [Sigma]). After centrifugation for 10 min at 14 000 ***g*** at 4 °C, the supernatants were recovered. The protein concentration was determined using a bicinchoninic acid protein assay kit (Pierce Biotechnology, Inc., Rockford, IL, USA). Samples containing equivalent amounts of protein were resolved by SDS/PAGE and transferred to polyvinylidene difluoride membranes. The membrane was probed with a primary antibody and subsequent HRP‐conjugated secondary antibody as recommended by the manufacturer of each antibody. Specific bands were visualized using Immobilon Western Chemiluminescent HRP Substrate (Merck Millipore, Billerica, MA, USA) and detected by a ChemiDoc Touch (Bio‐Rad, Hercules, CA, USA).

### Immunofluorescence

Cells plated onto Geltrex‐coated coverslips were fixed with 4% paraformaldehyde in PBS for 30 min at RT. The fixed cells were permeabilized and blocked with 0.3% Triton X‐100 with 2% bovine serum albumin in PBS for 30 min at RT. The cells were incubated with primary antibody against ACADVL overnight at 4 °C, washed with PBS, and incubated with a secondary Alexa 488‐conjugated antibody for 1 h at RT. Coverslips were mounted using VECTASHIELD Antifade Mounting Medium with DAPI (Vector Laboratories, Burlingame, CA, USA). Fluorescence images were acquired using a confocal laser‐scanning microscope (FLUOVIEW FV10i; Olympus, Tokyo, Japan).

### Measurement of cellular ATP levels

Cellular ATP concentrations were determined with a luciferin‐luciferase reaction using an ATP Bioluminescence Assay kit HS II (Roche, Penzberg, Upper Bavaria, Germany). Briefly, cells were harvested and resuspended in PBS to a concentration of 1 × 10^5^ cells·mL^−1^. A 50‐μL aliquot of each cell suspension was mixed with an equivalent volume of cell lysis buffer supplied with the kit. After 5 min incubation at room temperature, the luciferase regent was added to the cell lysate, and the solution was mixed by vortexing. Luminescence was measured using a Mini Lumat LB 9506 Single Tube (Berthold Technologies, Bad Wildbad, Germany), and the percentage of luminescence relative to control GSCs was determined.

### Measurement of mitochondrial membrane potential

Cells were incubated with MitoTracker Orange (final concentration 12.5 nm; Thermo Fisher Scientific) for 30 min at 37 °C. After cells were washed with PBS and harvested, the cells were resuspended in PBS and subjected to flow cytometry. Gating for single cells was established using forward scatter. At least 1 × 10^4^ cells were evaluated and gated using side and forward scatters to identify viable cell populations. All analyses were run on the FACSCanto II Flow Cytometer (BD Biosciences, San Jose, CA, USA), and the data were analyzed using flowjo software, version 7.6.5 (Treestar Inc., Ashland, OR, USA).

### Statistical analysis

All data are shown as the mean ± SD. Data were analyzed using a Student's *t*‐test for comparisons between two groups and a one‐way ANOVA followed by Dunnett's test for comparisons of more than two groups. Differences with a *P*‐value < 0.05 were considered statistically significant.

## Results

### Lico A specifically induces cell death in GSCs

**Figure 1:**
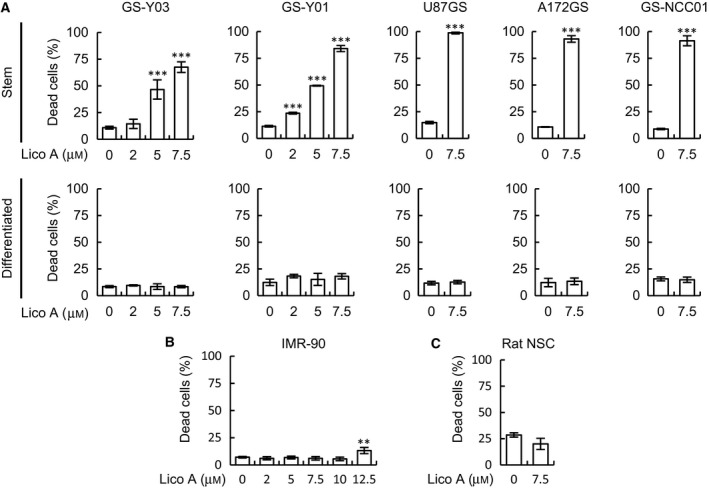
Licochalcone A (Lico A) specifically induces cell death in glioma stem cells (GSCs) but not in differentiated GSCs. GSCs (A, Stem, upper graph), serum‐differentiated GSCs (A, Differentiated, lower graph), IMR‐90 cells (B), and rat cortical neural stem cells (C, Rat NSC) treated with the indicated concentrations of Lico A for 6 days were subjected to a cell death assay using the trypan blue dye exclusion method. Next, the percentage of dead cells was determined. In (A–C), the values are presented as the mean ± SD from triplicate samples of a representative experiment repeated with similar results. **P < 0.01 and ***P < 0.001 vs. control‐treated cells.

**Figure 2:**
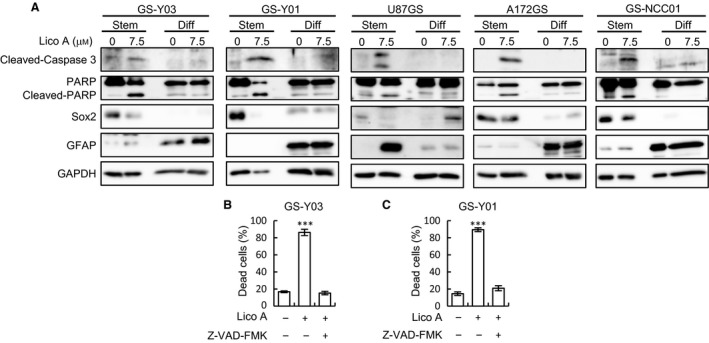
Licochalcone A (Lico A) treatment induces caspase‐dependent cell death in glioma stem cells (GSCs). (A) GSCs (Stem) and serum‐differentiated GSCs (Diff) treated without or with Lico A (7.5 μm) for 6 days were subjected to an immunoblot analysis of the indicated proteins. (B,C) GS‐Y03 (B) and GS‐Y01 (C) were treated with Lico A (7.5 μm) in the absence or presence of Z‐VAD‐FMK (50 μm) for 6 days. Next, the percentage of dead cells was determined using trypan blue. Values are presented as the mean ± SD from triplicate samples of a representative experiment repeated with similar results. ***P < 0.001 vs. control‐treated cells.

Lico A suppresses the proliferation, migration, and invasion of several human cancer cells 11, 12, 13; however, the effect of Lico A on glioblastomas and GSCs remains obscure. We first examined the cytotoxic effect of Lico A on glioblastoma stem cells. Lico A increased the percentage of dead GSCs in a concentration‐dependent manner (Fig. 1A, upper graphs, GS‐Y03 and GS‐Y01), and more than 65% of GSCs died after 6 days of exposure to 7.5 μm Lico A (Fig. 1A, upper graphs). In contrast, treatment with 7.5 μm Lico A did not induce cell death in differentiated GSCs (Fig. 1A, lower graphs). We next evaluated the toxicity of Lico A on IMR‐90 cells, human normal fibroblasts, and rat cortical NSCs (Rat NSCs). The viability of normal cells treated with Lico A at concentrations up to 10 μm remained unaffected (Fig. 1B,C), suggesting that Lico A specifically induces cell death in GSCs without affecting human normal fibroblasts and NSCs. We next investigated whether the caspase pathway was activated in Lico A‐treated GSCs. As shown in Fig. 2A, the processing of the cleaved form (active) of caspase 3 and the caspase substrate PARP were increased in GSCs but not in differentiated GSCs after Lico A treatment. To determine whether the observed cell death was caspase‐dependent apoptosis, we examined the effect of a pan‐caspase inhibitor on Lico A‐induced cell death. The pan‐caspase inhibitor Z‐VAD‐FMK fully inhibited Lico A‐induced cell death in GSCs (Fig. 2B,C), suggesting that Lico A induces apoptosis.

### Lico A induces the consecutive cleavage of caspase‐9 and caspase‐3

**Figure 3:**
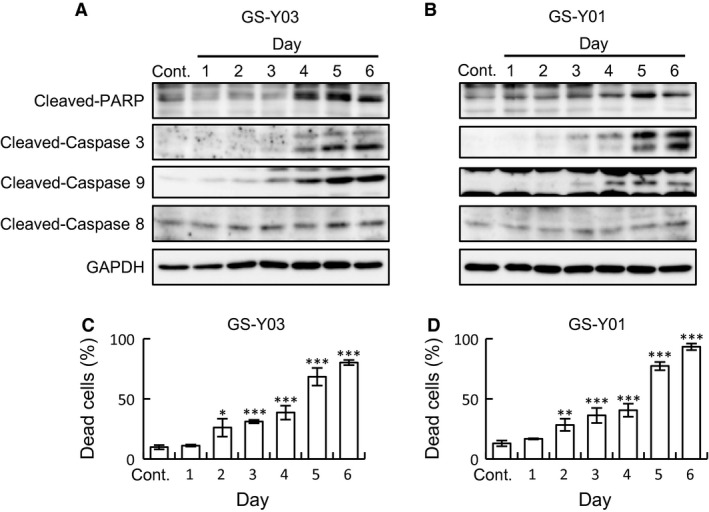
Licochalcone A (Lico A) triggers the caspase‐9‐dependent processing of caspase 3. GS‐Y03 (A) and GS‐Y01 (B) were treated without (Cont.) or with Lico A (7.5 μm) for the indicated time periods (1–6 days) and subjected to an immunoblot analysis of the indicated proteins. (C,D) Cells were treated as described in (A,B) and then subjected to a cell death assay using trypan blue. The percentage of dead cells was determined. In (C) and (D), the values are presented as the mean ± SD from triplicate samples of a representative experiment repeated with similar results. *P < 0.05, **P < 0.01, and ***P < 0.001 vs. control treated cells.

We next examined the time‐dependent activation of the caspase signaling pathway after Lico A treatment. The processing of caspase‐3 and PARP were distinctly detected in GS‐Y03 and GS‐Y01 after treatment with 7.5 μm Lico A for 4 and 5 days, respectively (Fig. 3A,B). Lico A treatment caused a time‐dependent increase in the proportion of dead cells and a coincident increase in the cleavage of caspase and PARP (Fig. 3C,D). Apoptotic cell death is typically categorized into the intrinsic or extrinsic pathway. We found that caspase‐9, an initiator caspase of the mitochondrial (intrinsic) apoptotic pathway, but not caspase‐8, an initiator of the extrinsic cell death pathway, was activated in Lico A‐treated GSCs in a time‐dependent manner (Fig. 3A,B). Notably, the cleaved form of active caspase‐9 was distinctly detected on days 3–4 after Lico A treatment prior to the production of cleaved caspase‐3. In contrast, Lico A treatment did not increase the cleavage of caspase‐8 (Fig. 3A,B). Thus, Lico A activates the mitochondrial death pathway but not the extrinsic apoptotic cell death pathway in GSCs. Furthermore, the delay between the start of Lico A treatment and the cleavage of caspase‐9, as well as the lack of effect on caspase‐8, suggests that Lico A does not directly activate initiator caspases.

### Lico A induces mitochondrial dysfunction in GSCs

**Figure 4:**
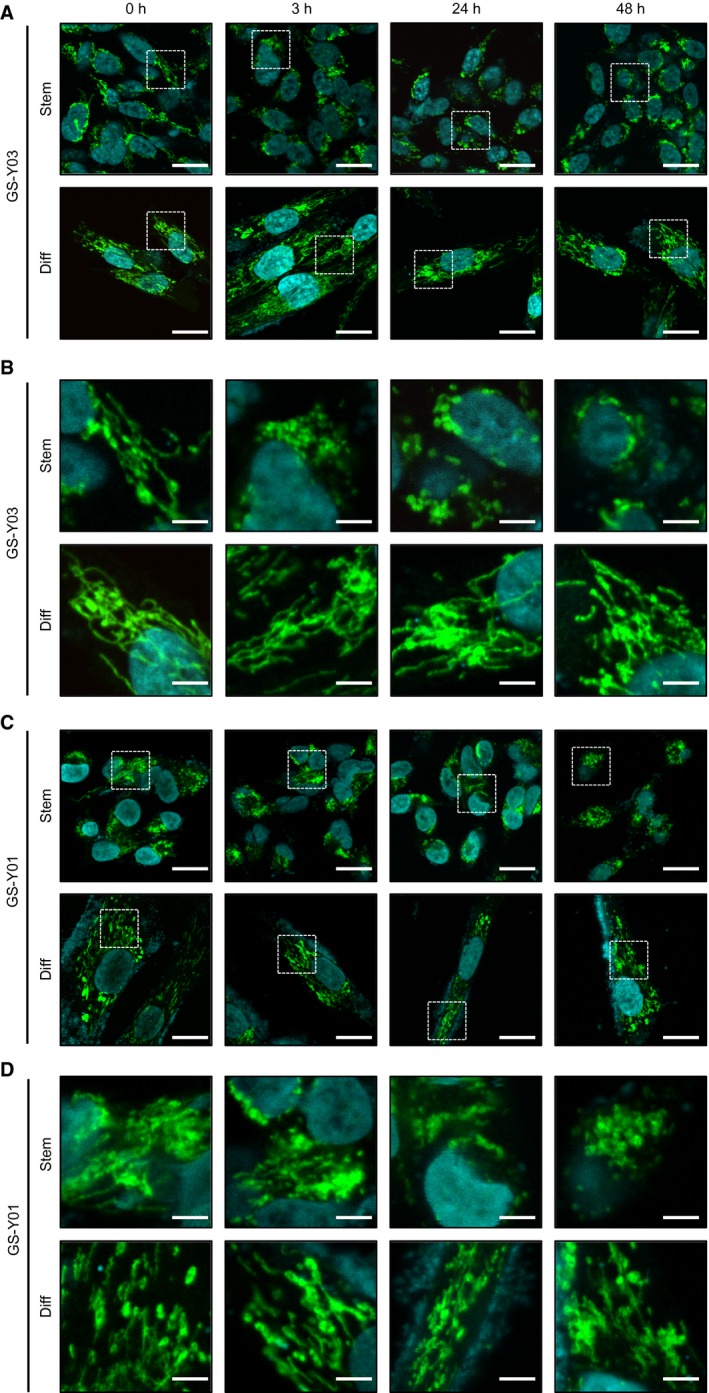
Licochalcone A (Lico A) specifically induces mitochondrial fragmentation in glioma stem cells (GSCs). GSCs (Stem) and differentiated GSCs (Diff) were treated with Lico A (7.5 μm) for the indicated times. Next, the cells were stained using an ACADVL (green) antibody and DAPI (blue). Photographs of the representative images are shown. (A,C): low magnification (scale bar = 20 μm), (B,D): high magnification (white squares in (A) and (C), respectively; scale bar = 5 μm).

**Figure 5:**
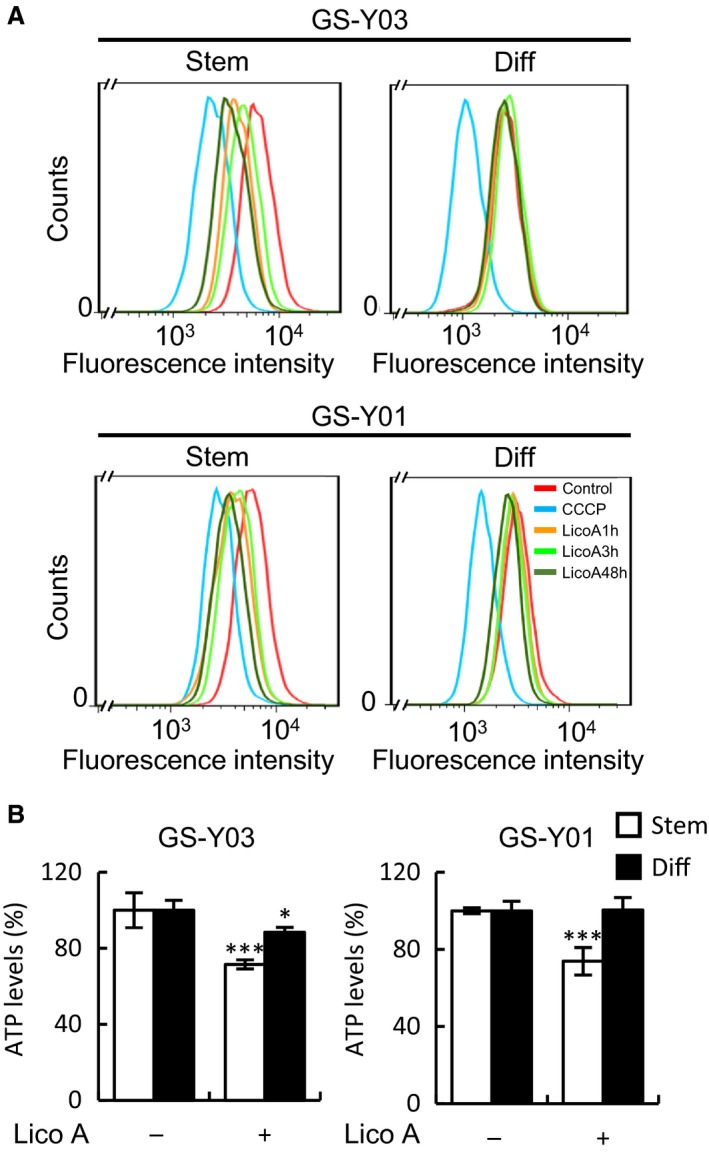
Licochalcone A (Lico A) induces mitochondrial dysfunction. (A) The indicated cells were treated without (Control) or with Carbonyl cyanide 3‐chlorophenylhydrazone (CCCP; 100 μm) for 1 h, or Lico A (7.5 μm) for the indicated time periods (1, 3, and 48 h). Next, the cells were stained with MitoTracker Orange, and the fluorescence intensity was quantified using flow cytometry. Representative flow cytometry histograms are shown. (B) Cells treated without or with Lico A (7.5 μm) for 24 h were subjected to the assay for ATP levels. Open bar, glioma stem cells (Stem); filled bar, differentiated‐GSCs (Diff). Values are presented as the mean ± SD from triplicate samples of a representative experiment repeated with similar results. *P < 0.05 and ***P < 0.001 vs. untreated control (–) cells.

**Figure 6:**
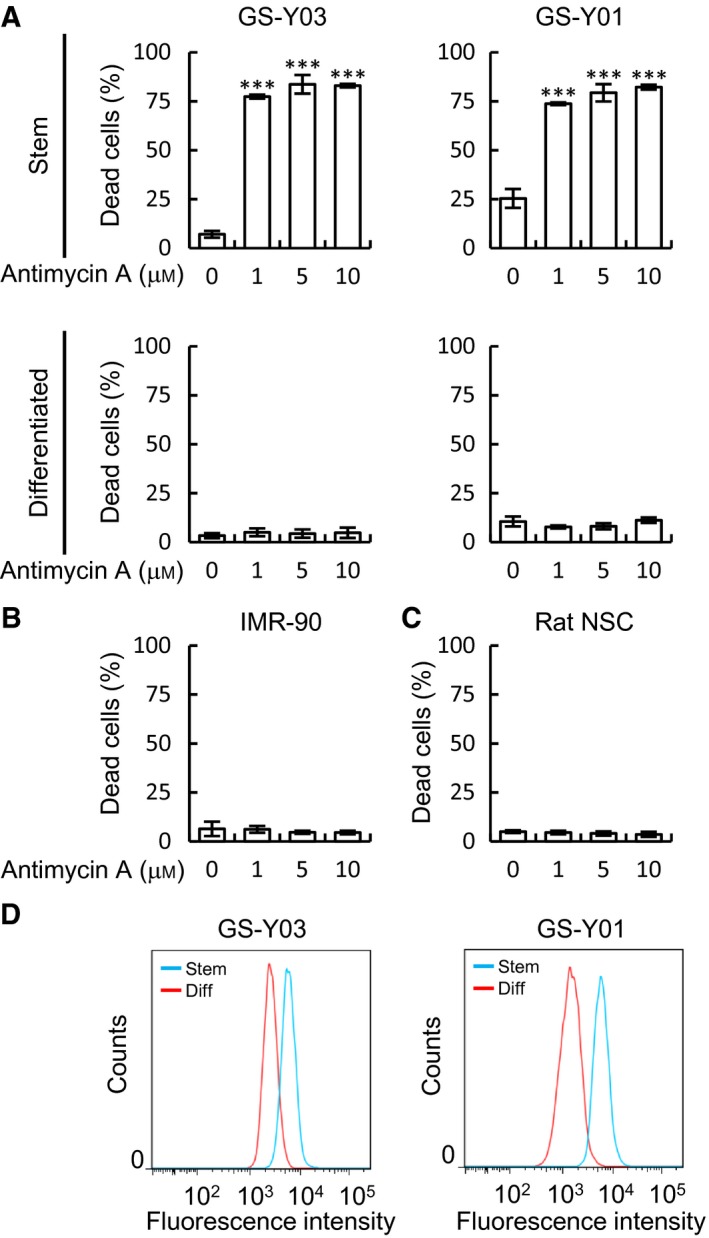
Inhibition of mitochondrial cytochrome *bc*_1_ complex induces cell death in glioma stem cells (GSCs) but not in differentiated GSCs. GSCs (A, Stem, upper graph), serum‐differentiated GSCs (A, Differentiated, lower graph), IMR‐90 cells (B), and rat cortical neural stem cells (C, Rat NSCs) treated with the indicated concentrations of antimycin A for 3 days were subjected to a cell death assay using the trypan blue dye exclusion method. Then, the percentage of dead cells was determined. In (A–C), the values are presented as the mean ± SD from triplicate samples of a representative experiment repeated with similar results. ***P < 0.001 vs. control treated cells. (D) GSCs and differentiated GSCs were stained with MitoTracker Orange, and the fluorescence intensity was quantified using flow cytometry. Representative flow cytometry histograms are shown.

Our results showed that Lico A strongly and specifically induced caspase‐9 activation prior to caspase‐3 cleavage in GSCs. Therefore, we predicted that Lico A treatment induced mitochondrial dysfunction via activation of the mitochondrial apoptotic pathway. We first assessed mitochondrial morphology after Lico A treatment using immunofluorescence staining for ACADVL (acyl‐CoA dehydrogenase, very long chain), a mitochondrial protein. Mitochondrial fragmentation was observed in GSCs after treatment with Lico A for 3 h, whereas mitochondrial fragmentation was not induced in differentiated GSCs even after treatment with Lico A for 48 h (Fig. 4). We next assessed whether mitochondrial dysfunction occurs concomitantly with mitochondrial fragmentation. We measured the mitochondrial membrane potential using MitoTracker Orange. The mitochondrial membrane potential showed an early decrease after Lico A treatment in GSCs (1 h treatment, Fig. 5A, Stem) but not differentiated GSCs (Fig. 5A, Diff). Next, we measured intracellular ATP levels in GSCs and differentiated GSCs after Lico A treatment. The intercellular ATP level in Lico A‐treated GSCs was significantly lower when compared with differentiated GSCs (Fig. 5B). In fact, it was previously reported that Lico A inhibits the mitochondrial cytochrome *bc*_1_ complex 8. Taken together, our results indicate that Lico A‐induced mitochondrial dysfunction results in the death of GSCs. Finally, we examined whether mitochondrial respiratory chain, particularly cytochrome *bc*_*1*_ complex, could be a convincing target for anti‐GSCs therapy. To this end, we evaluated the toxicity of antimycin A, a classical inhibitor of mitochondrial cytochrome *bc*_*1*_ complex. Similarly to Lico A, antimycin A strongly induced cell death in GSCs but not differentiated GSCs, IMR‐90, and NSCs (Fig. 6A–C). Additionally, we compared mitochondrial membrane potential between GSCs and differentiated GSCs under nontreated condition. The membrane potential of GSC is much higher than the differentiated GSCs (Fig. 6D). These data indicate that GSCs might rely on mitochondrial oxidative phosphorylation for energy production, suggesting that mitochondrial respiratory chain of GSCs could be a therapeutic target.

## Discussion

Lico A possesses anticancer effects against noncancer stem cells, such as nonsmall cell lung cancer cells, oral cancer cells, and cervical cancer cells 13, 23, 24; however, the effects of Lico A on cancer stem cells are unknown. We are the first to demonstrate that Lico A specifically induced cell death via mitochondrial dysfunction in GSCs but not differentiated GSCs.

Previous studies focused on the anticancer effects of high concentrations of Lico A (> 50 μm) 11, 12, 13; however, Lico A induced cell death in normal human fibroblasts (IMR‐90 cells), even at a concentration of 12.5 μm (Fig. 1B). Because high concentrations of Lico A affect both cancer cells and normal cells, Lico A should be used at low concentrations (< 12.5 μm) for clinical/preclinical applications. In contrast to previous reports, we found that low concentrations of Lico A specifically induced cell death in GSCs, and almost all GSCs died after a 6‐day treatment with 7.5 μm Lico A. Both normal fibroblasts and NSCs were not affected at these concentrations, suggesting that Lico A effectively targets cancer stem cells *in vitro*.

In our study, Lico A also specifically induced mitochondrial fragmentation and a decrease in both membrane potential and intracellular ATP levels in GSCs. Interestingly, Lico A induced mitochondrial dysfunction within 1 h of treatment in GSCs but not in differentiated GSCs (Fig. 5A). It is previously reported that Lico A inhibited the mitochondrial *bc*_1_ complex in both *Plasmodium falciparum* and rat liver 8. Thus, it is likely that Lico A also directly inhibits the mitochondrial respiratory chain in GSCs at low concentrations without cytotoxicity against normal cells. However, the mechanism of how GSCs are more sensitive to Lico A than differentiated GSCs remains unclear. One possibility is that the differences in components of respiratory chain complex could change the sensitivity of Lico A between GSCs and differentiated‐GSCs. For instance, Mi‐Ichi *et al*. reported that Lico A sensitivity of mitochondria of parasites is higher than rat liver because of the *bc*_*1*_ complex properties 8. It is unclear whether significant differences such as differences between parasites and mammals could be found between GSCs and non‐GSCs. However, further investigations are needed to search for the differences in mitochondria between GSCs and non‐GSCs. Another possibility is a drug elimination mechanism. It is generally accepted that the drug efflux activity of cancer stem cells is higher than that of noncancer stem cells. For example, breast cancer resistance protein 1, an ATP‐binding cassette (ABC) transporter associated with the export of temozolomide, is highly expressed in GSCs when compared with non‐GSCs 25. In contrast, it was recently reported that several types of ABC transporters are highly expressed in differentiated GSCs when compared with GSCs 26. Therefore, it is possible that Lico A is exported by these ABC transporters in differentiated GSCs. Further studies are needed to test this hypothesis.

Importantly, we found that Lico A induced mitochondrial dysfunction following depolarization in GSCs but not differentiated GSCs. It is well known that cancer cells produce most of their energy by aerobic glycolysis, that is, the Warburg effect. Recent studies showed that several types of cancer stem cells, including the GSCs, rely on oxidative phosphorylation for ATP production to survive 27, 28, 29, 30. Indeed, antimycin A, a classical inhibitor of mitochondrial respiratory chain, induces cell death in GSCs. In contrast, antimycin A was not toxic in normal fibroblast and NSCs even at a concentration of 10 μm (Fig. 6A–C). Moreover, mitochondrial membrane potential is higher than differentiated GSCs under nontreated condition (Fig. 6D). Based on previous reports and our present study, respiratory chain could be a specific and a sensitive therapeutic target for GSCs. Efforts are underway to develop drugs that target the mitochondria of cancer stem cells. For instance, Atovaquone, an FAD‐approved antimalarial drug, directly binds to Cytochrome *b*, a component of the Cytochrome *bc*_1_ complex. Atovaquone preferentially inhibits MCF7‐derived breast cancer stem cells 31. NV‐128, a derivative of isoflavone, induces Cytochrome *c* oxidase degradation and activates the cell death pathway in ovarian cancer stem cells 32. Lico A may be a novel mitochondria‐targeting drug for cancer stem cells; however, the effect of Lico A on other types of cancer stem cells remains unclear. Further investigation is needed to elucidate whether Lico A induces cell death in other types of cancer stem cells that rely on the mitochondrial oxidative phosphorylation for energy production. Additionally, it remains unknown if Lico A induces glioblastoma stem cell death *in vivo*. Notably, several previous papers reported that Lico A exerts anticancer effects without toxicity in mice 13, 33, 34. However, further investigation is needed to elucidate the effect of Lico A on GSCs using an intracranial xenograft model.

In summary, we demonstrated for the first time that Lico A decreases the mitochondrial membrane potential and intracellular ATP as well as induces mitochondrial fragmentation in GSCs. This mitochondrial dysfunction activates the mitochondrial apoptotic signaling pathway, thus resulting in cell death. Our findings suggest that Lico A can serve as a novel cancer stem cell‐targeting agent.

## Author contributions

KK, CK, and MO designed the research, analyzed the data, and wrote the paper; KK and MO performed the experiments; S. Suzuki, HS, HT, TS, and SS assisted with data interpretation. TK and YN provided patient materials.
